# Dose-Intensified Stereotactic Ablative Radiation for Localized Prostate Cancer

**DOI:** 10.3389/fonc.2022.779182

**Published:** 2022-02-21

**Authors:** Lily Chen, Bhavani S. Gannavarapu, Neil B. Desai, Michael R. Folkert, Michael Dohopolski, Ang Gao, Chul Ahn, Jeffrey Cadeddu, Aditya Bagrodia, Solomon Woldu, Ganesh V. Raj, Claus Roehrborn, Yair Lotan, Robert D. Timmerman, Aurelie Garant, Raquibul Hannan

**Affiliations:** ^1^School of Medicine, The University of Texas Rio Grande Valley, Edinburg, TX, United States; ^2^Department of Radiation Oncology, University of Texas Southwestern Medical Center, Dallas, TX, United States; ^3^Department of Population and Data Sciences, University of Texas (UT) Southwestern Medical Center, Dallas, TX, United States; ^4^Department of Urology, University of Texas Southwestern Medical Center, Dallas, TX, United States

**Keywords:** prostate, prostate cancer, SBRT (stereotactic body radiation therapy), stereotactic ablative radiation (SAbR), genitourinary (GU), dose-intense radiation therapy, gastrointestinal (GI), toxicity

## Abstract

**Purpose:**

Stereotactic ablative radiation (SAbR) has been increasingly used in prostate cancer (PCa) given its convenience and cost efficacy. Optimal doses remain poorly defined with limited prospective comparative trials and long-term safety/efficacy data at higher dose levels. We analyzed toxicity and outcomes for SAbR in men with localized PCa at escalated 45 Gy in 5 fractions.

**Methods and Materials:**

This study retrospectively analyzed men from 2015 to 2019 with PCa who received linear-accelerator-based SAbR to 45 Gy in 5 fractions, along with perirectal hydrogel spacer, fiducial placement, and MRI-based planning. Disease control outcomes were calculated from end of treatment. Minimally important difference (MID) assessing patient-reported quality of life was defined as greater than a one-half standard deviation increase in American Urological Association (AUA) symptom score after SAbR.

**Results:**

Two-hundred and forty-nine (249) low-, intermediate-, and high-risk PCa patients with median follow-up of 14.9 months for clinical toxicity were included. Acute urinary grade II toxicity occurred in 20.4% of patients. Acute grade II GI toxicity occurred in 7.3% of patients. For follow-up > 2 years (n = 69), late GU and GI grade ≥III toxicity occurred in 5.8% and 1.5% of patients, respectively. MID was evident in 31.8%, 23.4%, 35.8%, 37.0%, 33.3%, and 26.7% of patients at 3, 6, 12, 24, 36, and 48 months, respectively. The median follow-up for biochemical recurrence was 22.6 months with biochemical failure-free survival of 100% at 1 year (n = 226) and 98.7% for years 2 (n = 113) and 3 (n = 54).

**Conclusions:**

SAbR for PCa at 45 Gy in 5 fractions shows an encouraging safety profile. Prospective studies with longer follow-up are warranted to establish this dose regimen as standard of care for PCa.

## Introduction

Prostate cancer (PCa) is the most common non-cutaneous malignancy among men in the United States (1). Given the availability of multiple effective modalities for treating early-stage PCa, balancing side effects, reducing cost, and improving convenience with long-term disease control are important. Ultra-hypofractionated radiotherapy (RT) delivered using stereotactic ablative radiation therapy (SAbR) is now a standard RT modality that offers convenient and cost-effective treatment for PCa (2–5). Additionally, SAbR offers the potential radiobiological advantage of delivering high doses per fraction, which would be predicted to maintain or improve disease control without increasing toxicity of adjacent organs (6, 7). However, model-based dose conversions from long-established conventionally fractionated RT (CFRT) must be verified by empiric clinical data, which at times have challenged initial assumptions (8–10).

Despite guideline suggestions of 36.25–42.7 Gy in 5–7 fractions (National Comprehensive Cancer Network (NCCN) V2.2021), actual comparative data on ideal SAbR dose are sparse and suggest room for further improvement. One phase 3 randomized control trial in men with intermediate–high-risk PCa indicated that ultra-hypofractionated RT (42.7 Gy in 7 fractions) is non-inferior to dose-escalated conventionally fractionated RT (CFRT) (11), which itself is known to have worse biochemical control than CFRT with brachytherapy boost for intermediate- and high-risk patients (12). Similarly, posttreatment biopsy studies at 2 years after SAbR have shown strong dose response from 32.5 to 42.5 Gy/5–6 fractions, with 47.6% positive biopsy at 32.5 Gy declining to a still notable 10.9% rate at 40–42.5 Gy in a single-center series (13, 14). Data for escalation of SAbR dose >40 Gy to further improve efficacy are limited.

One prior prospective phase I–II SAbR dose-escalation study from our institution showed unacceptable rates of late high-grade rectal toxicity at the 50-Gy/5 fraction dose level (5, 15, 16). Since then, several approaches have been developed to mitigate such complications, such as using a perirectal hydrogel spacer (16–19). We performed a retrospective review of our single institutional experience treating PCa with SAbR in a larger cohort of 249 patients with 45 Gy in 5 fractions with standard prostate fiducials, hydrogel perirectal spacer use, and MRI-based planning.

## Methods

### Patients and Eligibility

45 Gy in 5 fractions is a standard of care at our institution for low-/intermediate-risk PCa following publication of our original Phase I/II experience (5, 15) and updated Phase II trial with hydrogel spaceOAR (20). An initial consultation is held, and applicable patient-specific treatment options are discussed including brachytherapy (high-dose rate and low-dose rate), external bean radiation therapy (conventional, moderate, stereotactic body radiation therapy (SBRT) fractionation), and combination of brachytherapy and external beam radiation therapy (EBRT). An appropriate decision is thus made after taking into consideration patient anatomy, baseline urinary symptoms, and disease characteristics. Using a tumor registry at a single tertiary care center, we identified 249 consecutively treated patients with localized PCa who had received 45 Gy/5 fraction SAbR between September 2015 and May 2019. Evaluated patients included men enrolled on a phase II clinical trial (n = 44) (17) or who were reviewed under an institutional review board-approved registry study. All patients underwent prostate fiducials and perirectal hydrogel spacer placement before SAbR. Androgen deprivation therapy (ADT) use and duration were at the discretion of the treating physician. Staging included baseline computed tomography (CT) or magnetic resonance imaging (MRI) (168 had MRI) of pelvis in all men, as well as bone scan at physician discretion based upon disease risk.

### SAbR Treatment Planning and Delivery

Treatment planning, dose constraints, simulation, setup, and treatment delivery parameters were conducted as previously reported (5, 15, 17). All patients were instructed to have a full bladder and empty their rectum with enemas at the time of CT simulation and treatment planning MRIs. Gold fiducial markers and a perirectal hydrogel spacer (21) were placed ≥5 days before simulation. Three fiducials were placed in the right lateral base, right apex, and left anterior mid gland. The hydrogel was injected transperineally on the posterior side of Denonvilliers’ fascia and the anterior rectal wall to minimize risk of pushing cancer cells away from a high-dose radiation field. Prophylactical but optional therapy included 4 mg of dexamethasone before each fraction of SAbR and use of alpha-blocker (i.e., tamsulosin) for at least the duration of treatment.

Treatment plans were created using fused thin-cut CT images (2 mm) and high-resolution MR images. The MR sequences included straight axial T2-weighted 2D fast spin echo (FSE) for the delineation of the prostate anatomy and straight axial T1-weighted spoiled gradient recalled echo (SPGR) that enhances the metal artifacts of fiducials allowing for CT to MRI image registration. The entire prostate constitutes the clinical target volume (CTV) for low-/intermediate-risk patients. A uniform expansion of 3 mm was added to the CTV to constitute the planning treatment volume (PTV). In cases where MRI showed seminal vesicle (SV) involvement or a lesion in the base of the prostate, CTV included proximal or full SV depending on involvement. Prior to reporting of our phase I toxicity data on 5-fraction 25-Gy pelvic SBRT nodal radiation (20), elective nodal RT for high-risk patients was not conventionally offered. Thus, this experience included only one patient who received elective nodal irradiation. The dose prescription for all men was 45 Gy in 5 fractions. Planning was performed with inversely planned IMRT with 95% PTV coverage by the prescription dose in a Linac-based platform, and 99% of PTV received a minimum of 90% of the prescription dose. The cumulative volume of all tissue outside of the PTV receiving a dose greater than 105% of the prescription dose should be no more than 15% of the PTV volume. The treating physician determined the anatomical delineations of the CTV, bladder wall, bladder trigone, rectum, sigmoid, bowel, penile bulb, femoral heads, and urethra. The bladder was contoured on a CT axial plane using its outer edge with a 5-mm inner depth from the outline defining the bladder wall. The rectal wall was defined as outer 5-mm circumference of the rectum. Prostatic urethra was identified within the prostate parenchyma on axial T2-weighted MRI sequencing in treatment position, where the inferior aspect of the prostatic urethra should coincide with the apex of the prostate and the superior aspect of the prostatic urethra coincides with the base of the prostate at the bladder inlet. Key institutional constraints were a) bladder wall D0.035 cc < 47.25 Gy, V18.3 Gy <18 cc; b) rectal wall circumference receiving 24 Gy < 50%, 39 Gy < 33%, D3cc < 50 Gy; c) urethra D0.035cc < 47.25 Gy, as summarized based on our prior trials (5, 15, 17). Planning constraints for the target and normal tissues used in this study are listed in **Table 1**.

**Table 1:** A. Prescription dose (45 Gy/5) and target coverage.

Average PTV D95% (% of Rx) ± standard deviation (range)	99% ± 1% of Rx (99%–105%)	

B. Dose volume constraints for normal tissues.	
*^a^* Bladder wall D (0.035 cc)		47.25 Gy (105% of Rx)
V Bladder (18.3 Gy) (cc)		18 cc
*^b^*% Rectal wall circumference (24 Gy)		50% of circumference
*^b^*% Rectal wall circumference (39 Gy)		33% of circumference
*^c^* Rectal wall D (3 cc)		50 Gy
*^d^* Prostatic urethra D (0.035 cc)		47.25 Gy (105% of Rx)

a Bladder wall was defined as the outer circumference of the visible bladder minus the inner lumen with a presumed wall thickness of 0.5 cm in all directions.

b % Rectal wall circumference was calculated by (axial length of rectal wall treated by 24 or 39 Gy) / (circumference). The axial length of the rectal wall treated by 24 or 39 Gy was defined as the level of mid-prostate gland by measuring the distance from 24- or 39-Gy isodose lines at the right and left edges of the rectal wall to the mid anterior rectal wall. Circumference of the rectum at mid-prostate level estimated using formula π * diameter.

c Peri-prostatic rectal wall was defined as the circumference of the rectum adjacent to the prostate.

d Prostatic urethra identified within the prostate parenchyma on axial T2-weighted MRI sequencing in the treatment position, where the inferior aspect of the prostatic urethra should coincide with the apex of the prostate and the superior aspect of the prostatic urethra coincides with the base of the prostate at the bladder inlet.

### Follow-Up

Prostate-specific antigen (PSA) and clinical status were generally assessed every 3–4 months for years 1–2, every 6 months for years 3–5, and every year starting at 5 years after treatment; however, this ultimately was according to patient and physician’s discretion.

### Toxicity Assessments

Toxicity was retrospectively assessed and defined using the National Cancer Institute Common Toxicity Criteria for Adverse Events (CTCAE) v. 5.0. Acute toxicity was defined as toxicity occurring less than 90 days from treatment. Late toxicity was defined as persistent or new toxicities occurring more than or equal to 90 days after treatment. Gastrointestinal (GI)/genitourinary (GU) treatment side effects were recorded through the most recent follow-up as of January 2020.

Patient-reported questionnaires [American Urological Association symptom score (also known as the International Prostate Symptom Score, IPSS) and Sexual Health Inventory for Men [SHIM] were collected before therapy and at each follow-up, as per standard. We quantified the proportion of patients reporting a minimally important difference (MID), defined as greater than a 1/2 standard deviation increase in IPSS at any given month after RT compared to pre-RT baseline (22). Continued baseline erectile dysfunction (ED) or worsening/new ED symptoms in patients after SAbR were recorded, as well as the date of symptom onset.

### Outcome Definitions

Overall survival (OS), PCa-specific survival (PCaSS), and biochemical failure-free survival (BFFS) were measured from radiation start date to event (death from any cause for OS, death from prostate cancer for PCaSS, biochemical failure, or death for BFFS) through the most recent follow-up as of September 2020. The Phoenix definition of nadir + 2 ng/ml after treatment constitutes a biochemical failure (23). PSA bounce was defined as an increase in PSA to nadir ≥0.2 ng/ml followed by subsequent and durable fall (24); these cases were not scored as BFFS events. The mean magnitude of PSA rise during bounce was calculated from when bounce first began. The median duration of PSA bounce was calculated from month of bounce until month of first recorded PSA decline.

### Statistical Analysis

Descriptive statistics were used to summarize baseline characteristics and cumulative acute and late GU and GI toxicities. The Kaplan–Meier method was used to estimate OS and BFFS. BFFS was calculated from completion of SAbR to biochemical failure or death. In the absence of these outcomes, patients were censored at last follow-up. Log-rank tests were conducted to investigate significant differences in OS and BFFS among NCCN risk groups.

## Results

A total of 249 patients were included. Demographic information is summarized in **Table 2**.

**Table 2:** Patient baseline characteristics.

	No.	%
**Toxicity follow-up, months**		
Median (IQR)	14.9 (9.5–25.4)	
**Outcome follow-up, months**		
Median (IQR)	22.6 (16.3–34.0)	
**Age, years**		
Median (range)	70 (51–94)	
**Prostate size cm^3^**		
Median (range)	41 (14–103)	
**AUA score**		
Median (range)	8 (0–29)	
**PSA**		
Median (range)	7.6 (0.8–24.7)	
**T-stage**		
T1c	192	77.11%
T2a	23	9.24%
T2b	13	5.22%
T2c	17	6.83%
T3a	4	1.61%
**Gleason**		
6 (3+3) 37		14.86%
7 (3+4)	146	58.63%
7 (4+3)	60	24.10%
8 (4+4)	4	1.61%
9 (4+5)	2	0.80%
**NCCN risk**		
Low	22	8.84%
Favorable Intermediate	93	37.35%
Unfavorable Intermediate	120	48.19%
High	13	5.22%
Metastatic	1	0.40%
**ADT use**		
All patients	73	29.32%
Low risk patients	1	4.55%
Favorable intermediate risk patients	11	11.83%
Unfavorable intermediate risk patients	50	41.67%
High risk patients	10	76.92%
Metastatic	1	100.00%
**ADT duration**		
<6 months	18	25.00%
6 months	45	62.50%
12–24 months	9	12.50%

AUA, American Urology Association; NCCN, National Comprehensive Cancer Network; ADT, Androgen Deprivation Therapy.

### Biochemical Outcomes

**Figure 1:**
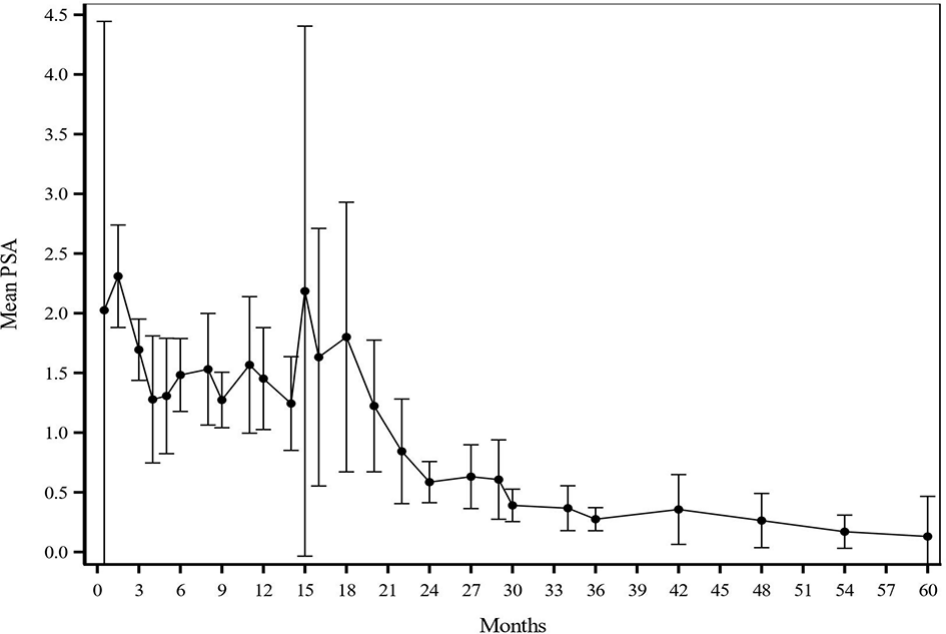
Mean PSA values at each given timepoint after treatment. PSA values were assessed generally every 3-4 months for year 1-2, every 6 months for years 3-5, and every year starting at 5 years after treatment. After SAbR, the median PSA nadir was 0.7 and 0.4 at 1-year and 2-year follow-up, respectively.

**Figure 2:**
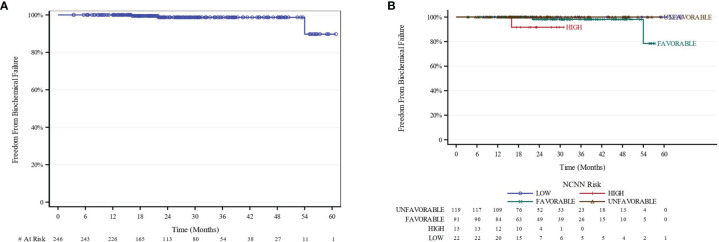
BFFS Kaplan-Meier curves **(A)** All patients. **(B)** Stratified by NCCN Risk. BFFS of 100% and 98.7% at 1 year and 2 years, respectively. BFFS of 100% for LR, 98.1% for favorable-intermediate, 100% for unfavorable-intermidiate, and 91.7% for HR patients at 2 years. Because no biochemical events were reported in the “low-risk” and ‘unfavorable-intermediate risk” NCCN risk group, they were excluded from analysis. BFFS, biochemical failure-free survival; NCCN, National Comprehensive Cancer Network.

The median follow-up for biochemical recurrence was 22.6 months (IQR 16.3–34.0). Seventy-three patients (29.3%) received ADT. The median pretreatment PSA for the entire cohort was 7.6 ng/ml (range 0.8–24.7). While premature to report, the trend so far shows a median PSA nadir of 0.7 and 0.4 at the 1- and 2-year follow-ups, respectively (**Figure 1**). Among patients who received ADT, the median PSA nadir at both the 1- and 2-year follow-ups showed a trend toward 0.05. Among patients who did not receive ADT, the median PSA nadir value is trending toward 0.9 and 0.6 at the 1- and 2-year follow-ups, respectively. PSA bounce was noted in 83 patients (33.3%) with a median time of 9 months to 1st bounce after SAbR (range, 3.0–30.0 months). Of the 83 patients who had PSA bounce, 15.7% (13 patients) had received ADT with SAbR. The mean magnitude of PSA rise during a bounce was 0.99 (SD 1.28; range 0.07–9.25). The median duration of PSA bounce was 4 months (range, 1.0–30.0 months). Of the 83 patients who had ≥1 PSA bounce, 70 (84.3%) had a PSA bounce detected within a 12-month period after RT, while the remainder exhibited PSA bounce after 12 months. BFFS was stable 98.7% at 2 years, as well as for 3 and 4 years (**Figure 2**). BFFS rates were 100% for low-risk patients (N = 22), 98.1% for favorable intermediate-risk patients (N = 91), 100% for unfavorable intermediate-risk patients (N = 119), and 91.7% for high-risk patients (N = 13) at 2 years (**Figure 2**).

We observed 3 biochemical failures; 2 patients had the favorable-intermediate risk disease, and 1 patient had high-risk disease. One local failure and 2 distant failures were reported.

### Survival

While clearly premature to report, OS at 2 and 3 years were 98.8% and 97.2%, respectively. PCaSS was 100% at 3 years. One high-risk patient with biochemical failure showed evidence of metastasis to the spine and lung on PSMA PET scanning 12 months after SAbR.

### Toxicity

The median follow-up for clinical toxicity was 14.9 months (IQR 9.5–25.4). Acute and late toxicities are reported in **Table 3**. Acute grade II GU toxicity was observed in 20.4% of patients and late grade II GU toxicity in 35.3% of patients (**Table 3A**). Patients who received any additional medical management, including urinary catheter, irrigation, or initiation of prescription medications for any period of time after RT, were designated grade II GU toxicity. No acute-grade ≥ III GU or GI toxicity events were observed. Late grade ≥ III GU toxicity was noted in 5 patients (2.1%). For patients with follow-up > 2 years (n = 69), late GU and GI grade ≥ III toxicity occurred in 5.8% and 1.5% of patients, respectively (**Table 3B**).

**Table 3:** Highest reported acute and late toxicities from the start of treatment.

(A)								
	All patients							
	Genitourinary				Gastrointestinal			
Grade	No.	%	No.	%	No.	%	No.	%
0	45	18.37	76	31.15	170	68.55	209	83.94
I	150	61.22	77	31.56	60	24.19	31	12.45
II	50	20.41	86	35.25	18	7.26	7	2.81
III	0	0	5	2.05	0	0	1	0.4
IV	0	0	0	0	0	0	1	0.4
**(B)**								
	>2 years follow-up				>3 years follow-up			
	Late GU		Late Gl		Late GU		Late GI	
Grade	No.	%	No.	%	No.	%	No.	%
0	11	15.94	50	72.46	7	21.88	22	68.75
I	20	28.99	15	21.74	8	25	7	21.88
II	34	49.28	3	4.35	15	46.88	2	6.25
III	4	5.8	1	1.45	2	6.25	1	3.13
IV	0	0	0	0	0	0	0	0

(A) All patients. (B) Stratified by follow-up years. At a median follow-up of 14.9 months, acute urinary grade II toxicity occurred in 20.4% of patients. Acute grade II GI toxicity occurred in 7.3% of patients. Late grade ≥ II GI toxicity occurred in 3.6% of patients. One patient had grade III (0.4%) GI toxicity, and another had grade IV GI toxicity (0.4%). No acute grade ≥ III GU or GI toxicity was noted. (A). For follow-up > 2 years (n = 69), late GU and GI grade ≥ III toxicity occurred in 5.8% and 1.5% of patients, respectively (B).

GU, genitourinary; GI, gastrointestinal.

Three of the five patients with late grade III GU toxicity developed urinary complaints requiring daily self-catheterization; of these, one patient needed additional botulinum toxin injection. Another patient developed recurrent gross hematuria with major clots and terminal dysuria eventually requiring TURP and hyperbaric oxygen treatment. The 5th patient’s urinary symptoms were suspected to be secondary to prostatitis; thus, he was treated with Medrol dose pack.

Late grade ≥ III GI toxicity was detected in 2 patients (0.8%). One patient with late grade III GI toxicity reported rectal pain and rectal bleeding with bowel movements 180 days after treatment. MRI revealed proctitis, and anoscopy showed an area of rectal telangiectasia without evidence of abscess or fistula. Colonoscopy revealed a large 3-cm anorectal ulcer involving 33% of anal circumference; biopsy confirmed radiation proctitis.

One patient with concomitant late grade III GU toxicity developed late grade IV GI toxicity. The patient’s condition was complicated by unusual and severe grade IV rectal ulcer that required diversion at ~6 months and subsequent rectourethral fistula, ultimately requiring abdominal perineal resection and cystoprostatectomy with diversion of both urine and bowel. Pathology reports showed adenocarcinoma with marked radiation-induced changes at the urethra margin without extraprostatic disease or seminal vesicle invasion. This case was notable for marked rectal wall infiltration of hydrogel spacer; given the timeline of the symptom onset correlating to expected resolution time of spacer gel, there may have been an interaction between RT injury and spacer gel infiltration that is not fully understood, as described in more detail in the published case report for this event (25).

### Quality of Life

Quality of life (QoL) data were assessed using the AUA IPSS, and sexual function was assessed using the SHIM questionnaire. No significant trend was observed with AUA and SHIM, which may be related to lack of patient compliance with questionnaires. Two hundred and forty seven patients completed the AUA IPSS questionnaire at baseline with 148, 111, 81, and 46 patients that was completed at 3, 6, 12, and 24 months, respectively. Two hundred and forty eight patients completed the SHIM questionnaire at baseline with 143, 97, 79, and 43 patients who completed them at 3, 6, 12, and 24 months, respectively. Overall, improvement in patient-reported urinary and sexual function symptoms was noted.

**Figure 3:**
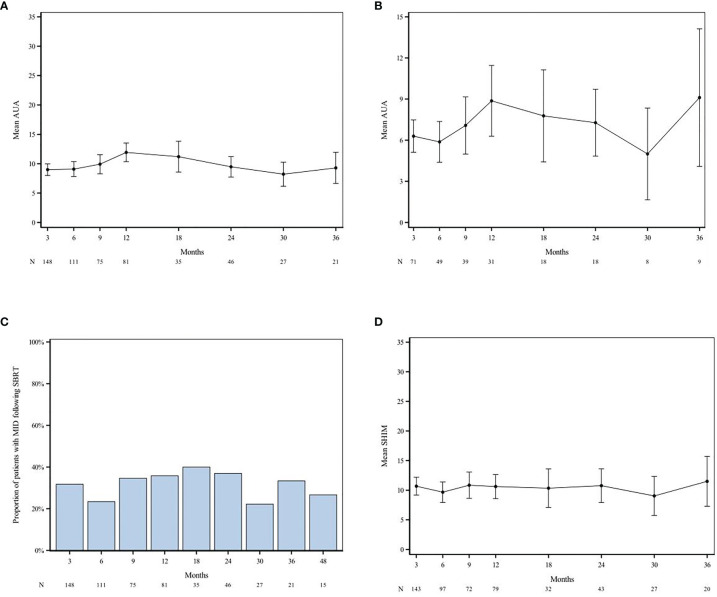
AUA and SHIM scores over time after SAbR. AUA trend amongst all patients **(A)** and those with low baseline AUA [AUA < 7; **(B)**] shows worse outcomes at 12 months +/- 3 months compared to baseline. Difference in MID GU was evident for 31.8%, 23.4%, 35.8%, 37.0%, 33.3%, 26.7% of patients at 3 months, 6 months, 12 months, 24 months, 48 months, respectively **(C)**. SHIM scores show decline at 6 months post-RT with subsequent plateau and increase (better) sexual function scores after 30 months **(D)**. “N” denotes the number of questionnaires completed at each time point. AUA, American Urological Association; SHIM, Sexual Health Inventory for Men; MID, Minimally Important Difference.

Half of the patients (48.6%) had moderate to severe lower urinary tract symptoms before treatment (baseline AUA ≥ 8) with a median baseline AUA symptom score of 7 (IQR 3.0–12.0). Mean AUA scores increased until 12 months after RT and subsequently declined until month 30 (**Figure 3A**). The AUA trend shows worse outcomes at 12 months +/- 3 months than at baseline; this trend was also noted among patients with low baseline AUA scores (AUA < 7) (**Figures 3A, B****)**. The AUA symptom score was also used to determine whether a patient had MID at any given month after RT. The MID GU score difference was evident for 31.8%, 23.4%, 35.8%, 37.0%, 33.3%, 26.7% of patients at 3, 6, 12, 24, 36, and 48 months, respectively (**Figure 3C**).

Half of the patients (53.6%) had erectile dysfunction (ED) before treatment (baseline SHIM ≤ 17) with a median baseline SHIM of 16.5 (IQR 5.0–23.0). The mean SHIM scores declined at month 6 with subsequent plateauing of scores for 9–30 months and increased (better) sexual function scores after month 30 (**Figure 3D**). Regardless of baseline potency, ED grade III was reported in 3 men, one of which had baseline grade III ED. The other two patients continued to experience a decrease in erectile function, despite use of medication, vacuum pump, or penile implantation; the median time to onset was 98 days. Of the men that were potent before SAbR (SHIM > 17) and those for which follow-up SHIM scores are available, 17.4%, (20/115) and 11.3% (13/115) maintained potency at 1 and 2 years after treatment, respectively. Of the baseline potent men, 24.3% (28/115) received ADT treatment and 20.0% (23/115) were on ED medications before RT.

## Discussion

SAbR has become increasingly used for managing men with localized PCa, but the optimal dose remains undefined. Posttreatment biopsy and comparative clinical trial data suggest room for further disease control improvement at doses >40 Gy in 5 fractions, with limited toxicity data (13, 14). Brachytherapy boost treatments have shown that escalating the biological dose of RT can improve prostate cancer outcomes; however, this modality consists of invasive treatments associated with increased toxicity and technical requirements (26–28). SAbR offers a promising and non-invasive alternative to brachytherapy for improving localized PCa outcomes. We report the largest study of early safety and outcomes of 45 Gy/5 fraction SAbR for localized PCa.

Our results indicate comparable GU toxicity with published results of patients treated with conventionally fractionated or moderately hypofractionated RT (29–35). In our study, 20.4% of patients reported acute grade II GU toxicity. Our findings were similar with observations reported by Catton et al. where acute grade II GU toxicities were observed in 27% with intermediate-risk PCa for hypofractionated RT of 60 Gy in 20 fractions (33). However, given the retrospective nature of our study, acute grade II GU toxicity is probably underreported because many patients are not seen back until the end of the 3-month period after treatment. Increased late grade II GU events in our study (35.3%) may be attributed to a lack of validated GU constraints for SAbR and to our use of higher SAbR dose, as suggested by the meta-analysis by Jackson et al. (36). Similarly, it is also important to acknowledge the high late grade II GU toxicity of 49.3% at the >2-year follow-up and 46.9% at the >3-year follow-up. Zelefsky et al.’s study with SBRT dose ranges from 32.5 Gy to 40 Gy in 5 fractions show increasing incidence of late grade II GU toxicities of 23.3%, 25.7%, 27.8%, and 31.4% for sequentially escalating dose levels of 32.5, 35, 37.5, and 40 Gy, respectively (14). Further analysis in Zelefsky’s study show that the 40-Gy dose group has significantly higher IPS scores at 12 and 24 months compared to lower-dose cohorts; however, at 36 months, there is no significant difference in IPS scores between the various dose cohorts (14). This trend of higher incidence of late grade II GU toxicity seen with higher doses provides some clarity for the high late grade II GU toxicity in this experience with 45 Gy in 5 fractions, along with reassurance for the gradual resolution of symptoms in the longer follow-up period. Late grade ≥ III GU toxicity in our cohort was 2.1%, which is consistent with 2.2% reported by Catton et al. Of note, toxicity data collection in this study ended in January 2020, and one patient with grade III GU toxicity ended up developing grade IV GU toxicity in March 2020, which was published as a case report (25). Given our short toxicity follow-up, toxicity is probably underreported. The percentage of patients who had MID was found to reach the lowest value at 6 months, followed by a plateau between 9 and 24 months and a relative decline after 30 months from RT. This finding is similar to that of another study of 35–36.25 Gy SAbR delivered in 5 fractions, in which the proportions of patients who reported a clinically significant decline in EPIC urinary scores were 34%, 40%, and 32.8% at 6, 12, and 36 months, respectively (37). The mechanisms of GU toxicity are multifactorial and therefore complex. On the one hand, they involve patient-related factors such as baseline urinary symptoms, benign prostatic hypertrophy, and ongoing medications (i.e., 5-α reductase inhibitors, α-1 blockers, etc.). On the other hand, they involve radiation delivery technique, bladder filling during treatment, dosimetric factors of the bladder wall, trigone, and urethral dose (38–40). While our institutional constraints and technique performed reasonably well, the exact dosimetric constraints to prevent GU toxicity are not well known yet and are actively being investigated.

The use of hydrogel spacer treatment before SAbR, along with derived rectal constraints from our phase I/II trial experience, probably contributed to lower risks of rectal toxicity in our patient subset than in previous reports (5, 16). Our patients had reduced acute and late grade GI ≥ II toxicities of 7.3% and 3.6%, respectively, as compared to 16.7% and 8.9%, respectively, as reported by Catton et al. (33). Another study reported late grade II rectal toxicity in 5% of patients when treated with SAbR of 36.25 Gy/5 fractions (41). Interestingly, our present study reports lower late grade ≥ II GI toxicity than our recently reported outcome of 14.3% (17), which may be related to improvements in technique of spacer placement since that trial preceded the rest of the patients in this study.

The focus of this study is safety as the short follow-up precludes any conclusion regarding the BFFS, which in our study was 98.7% at the 2-, 3-, and 4-year follow-ups. Dose escalation in multiple studies has shown improved BFFS in patients treated with escalating SAbR doses of 40 Gy or higher in 5 fractions than with lower doses, showing a faster PSA decline with dose escalation (14, 19, 42–44). These findings, while premature to conclude, are so far trending in a similar direction as reported in Zelefsky et al.’s 5-year outcomes of dose escalation, which reported BFFS trends of 83%, 85%, 90%, and 98% and lower PSA nadir values at 2 years of 0.7, 0.59, 0.46, and 0.48, respectively, in their 32.5, 35, 37.5, and 40 Gy in five fraction treatment arms, respectively, which was confirmed with declining rates of positive biopsy (14). In our study, a larger proportion of unfavorable intermediate-risk patients received ADT (41.8% vs. 11.8%) than favorable intermediate-risk patients, which confounds the biochemical response and PSA kinetics. The high percentage of patients who received ADT in the unfavorable intermediate-risk category may help to explain the paradoxical improvement of BFFS in this cohort. It also adds to available evidence that ADT improves SAbR outcome for this patient population (45).

Previously reported studies have shown a faster PSA decline with SAbR as compared to other fractionations and on par with brachytherapy (24, 42–44, 46). PSA nadir dose response trends have been shown to predict long-term biochemical control of PCa with follow-ups beyond 5 years (47). Thus, given our short median outcome follow-up, we are not able to draw any conclusions regarding our PSA kinetics data. However, our findings so far are trending in the correct direction. We will continue to monitor this trend. In the setting of already seeded distant metastasis at the time of SAbR, it may allow faster detection/discovery of the distant metastasis allowing for metastasis directed therapy eventually leading to better outcome for PCa patients (48, 49). One hindrance to the early detection of failure is the observed PSA bounce with SAbR. In our cohorts, approximately one-third of the patients experienced PSA bounce at a median time of 9 months with a mean increase of 1.0 that lasted a median duration of 4 months. These parameters should guide clinicians in distinguishing a PSA bounce from biochemical failure.

Our study was limited by short follow-up, single institutional experience, and the retrospective nature of the analysis that is prone to bias. The short follow-up precludes any conclusions on outcome and limits conclusions on late toxicity which may be underreported. It is important to note that our study contains a high percentage of low-favorable intermediate risk PCa patients (~45%) where a moderate-dose regimen (e.g., 34 Gy/5 fractions) has shown high efficacy while minimizing specifically grade II GU toxicity (50). Additional limitations include the lack of account for certain baseline factors, such as using anticoagulation, which could provide an explanation to patients developing rectal bleeding. The attrition of the QoL questionnaire response over time is also a concern that can affect the validity of patient-reported analysis.

This is one of the largest retrospective series that report the side effects of 45 Gy in 5-fraction SAbR for localized PCa with the use of a perirectal hydrogel spacer and MRI-based planning in a linear accelerator, indicating the feasibility of this strategy. The results show that 45 Gy in 5-fraction SAbR for PCa has acceptable GI and GU side effects using a perirectal hydrogel spacer along with appropriate constraints and technology. Longer follow-up is required to adequately assess late toxicity and outcome.

## Data Availability Statement

The original contributions presented in the study are included in the article/supplementary material. Further inquiries can be directed to the corresponding author.

## Ethics Statement

The studies involving human participants were reviewed and approved by the UT Southwestern Human Research Protection Program. Written informed consent for participation was not required for this study in accordance with the national legislation and the institutional requirements.

## Author Contributions

LC and BG led the data collection, literature search/review, and authoring text. RH led the study design and research question ND, MF, MD, JC, AB, SW, GR, CR, YL, RT, and AuR were collaborators for the editorial feedback. AnG and CA collaborated in the statistical analysis. All authors contributed to the article and approved the submitted version.

## Conflict of Interest

The authors declare that the research was conducted in the absence of any commercial or financial relationships that could be construed as a potential conflict of interest.

## Publisher’s Note

All claims expressed in this article are solely those of the authors and do not necessarily represent those of their affiliated organizations, or those of the publisher, the editors and the reviewers. Any product that may be evaluated in this article, or claim that may be made by its manufacturer, is not guaranteed or endorsed by the publisher.
